# Case Report: Investigation and molecular genetic diagnosis of familial hypomagnesaemia

**DOI:** 10.12688/f1000research.19006.2

**Published:** 2019-12-05

**Authors:** Jamie Willows, Maryam Al Badi, Chloe Richardson, Aisha Al Sinani, Noel Edwards, Sarah Rice, John A. Sayer

**Affiliations:** 1 Renal Services, The Newcastle upon Tyne Hospitals NHS Foundation Trust, Newcastle upon Tyne, NE7 7DN, UK; 2 National Diabetes and Endocrine Center, Royal Hospital, Ministry of Health, Muscat, Oman; 3 Institute of Genetic Medicine, Newcastle University, Central Parkway, Newcastle upon Tyne, NE1 3BZ, UK; 4 NIHR Newcastle Biomedical Research Centre, Newcastle University, Newcastle upon Tyne, NE4 5PL, UK

**Keywords:** hypomagnesaemia, with secondary hypocalcaemia, TRPM6, molecular genetics

## Abstract

Genetic mutations causing familial hypomagnesaemia syndromes are well-recognised.  Affected patients can present with severe symptoms of hypomagnesaemia, such as seizures or cardiac arrhythmia.  We report an affected child, from a consanguineous family, who presented in the first weeks of life with seizures secondary to hypomagnesaemia, without other associated clinical features.  We performed whole exome sequencing in the affected child and segregation analysis within the family, which revealed a novel homozygous missense mutation in *TRPM6*, which was confirmed as a heterozygous allele in both parents and two younger siblings who had transient hypomagnesaemia. Using *in silico* modelling, we provide evidence that the missense variant p.(K1098E) in *TRPM6 *is pathogenic, as it disrupts stabilising TRP domain interactions. Management of familial hypomagnesaemia relies on prompt recognition, early magnesium replacement and lifelong monitoring.

## Introduction

Homeostasis of the serum magnesium level is essential for human cellular function, and levels are maintained in the normal range by tight control of magnesium reabsorption by the kidney tubules ^1^. Hypomagnesaemia can manifest with a range of symptoms, from tremor, muscle spasms or nystagmus through to seizures, arrhythmias and cardiac arrest. Early identification of the electrolyte abnormality is vital, as treatment with magnesium replacement is efficacious and inexpensive. Common causes of hypomagnesaemia in adults include refeeding syndrome, diarrhoea, malabsorption, alcohol abuse and medications such as proton pump inhibitors ^2, 3^. Renal magnesium wasting is indicated by an inappropriately high fractional excretion of magnesium in urine despite hypomagnesaemia, and is seen in post-obstructive diuresis, the recovery phase of acute tubular necrosis, hypercalcaemia and in response to certain diuretics ^4^. However, genetically inherited mutations that cause renal hypomagnesaemia are well-recognised, and typically present in childhood if they are secondary to autosomal recessive disorders ^5^. Genetic forms of hypomagnesaemia should also be considered in certain clinical scenarios, such as in the presence of a positive family history of related disorders, consanguinity, or fulminant presentation. 

Once a genetic cause of hypomagnesaemia is suspected, work-up can be guided by associated features and age at presentation. Though obtaining a genetic diagnosis will not alter the treatment of magnesium replacement therapy, it is vital for identifying others at risk and family counselling, and may help to guide the clinician to screen for associated phenotypic features.

## Case report

We report a child from a consanguineous family (parents were second cousins) from Oman, who presented with seizures and hypomagnesaemia. The affected individual, a female child, presented at 20 days of age with tonic-clonic seizures. There was no history of fever or diarrhoea, and after an uncomplicated pregnancy she had been born healthy at term, without syndromic features. Serum magnesium was severely low at 0.35 mmol/L and was associated with a mild hypocalcaemia and suppressed parathyroid hormone (PTH) ( Table 1). The urinary fractional excretion of magnesium was inappropriately in the normal range given the severe degree of hypomagnesaemia present, suggesting contributory renal magnesium wasting. There were no other specific clinical or biochemical features; of note peripheral oxygen saturations and capillary blood glucose levels were within normal limits. Renal ultrasound scan was normal, with no nephrocalcinosis. She was initially treated with intravenous magnesium (20% MgCl _2_ 0.1 mmol/kg every 6 hours p.r.n.) and calcium replacement (10% Calcium Gluconate 0.11 mmol/kg). At 4 years of age she is now supported with high-dose oral magnesium supplements (magnesium sulphate 500mg qds) alone, and remains well with no further seizures, though she maintains a low serum magnesium level between 0.4–0.6 mmol/L.

**Table 1.** Clinical and biochemical features of siblings.

Child	Age at diagnosis / screening	Clinical Presentation	Serum Magnesium at presentation (NR 0.65–1.1 mmol/L)	Serum calcium (corrected) at presentation (NR 1.7–2.8 mmol/L)	Parathyroid hormone (NR (NR 1.6–6.9 pmol/L)	Fractional Excretion of Magnesium (NR 2–4%)	Current serum magnesium (NR 0.65–1.1 mmol/L)	Maintenance Magnesium supplements (magnesium sulphate)
II:1	20 days	Generalised seizure	0.35	1.38	1.11	2.1%	0.4–0.6 age 5 years	500 mg qds
II:2	18 days	Complex partial seizure	0.53	2.62	5.6	5.5%	0.7–0.9 aged 3 years	300 mg bd reduced to none
II:3	7 days	Asymptomatic	0.6	2.60	N/A	N/A	0.7–1.0 aged 1 year	None

NR, normal range; Fractional excretion of magnesium (%) = Urine Magnesium × Plasma Creatinine / (0.7 × Plasma Creatinine × Urine creatinine) × 100.

Of note, a younger sibling of the proband, also female, presented at 18 days old with abnormal eye movements thought to be as part of a complex partial seizure. Her serum magnesium was below normal limits (0.53 mmol/L), with serum calcium and PTH within the normal range ( Table 1), and normal blood glucose. The fractional excretion of magnesium was inappropriately high, and again renal ultrasound scan was normal and no other clinical features were noted. She was treated with intravenous magnesium replacement (20% MgCl _2_ 0.1 mmol/kg every 6 hours p.r.n.). Eye movements normalised post magnesium treatment and did not recur, though it remains impossible to be certain of a causal relationship between the hypomagnesaemia and the neurological presentation. She was then treated with a period of maintenance oral magnesium replacement (magnesium sulphate 300 mg b.d.). At 2 years of age she remains well with no further seizures, and she maintains magnesium levels within the normal range without additional supplementation. A younger asymptomatic male sibling was screened with serum biochemistry tests at 1 week of age. Serum magnesium was low at 0.6 mmol/L, with normal serum calcium and PTH levels ( Table 1). Supplementation was not started, and by 1 year of age serum magnesium was within the normal range. Both parents have been shown have normal serum magnesium levels.

## Genetic investigations

**Figure 1.**
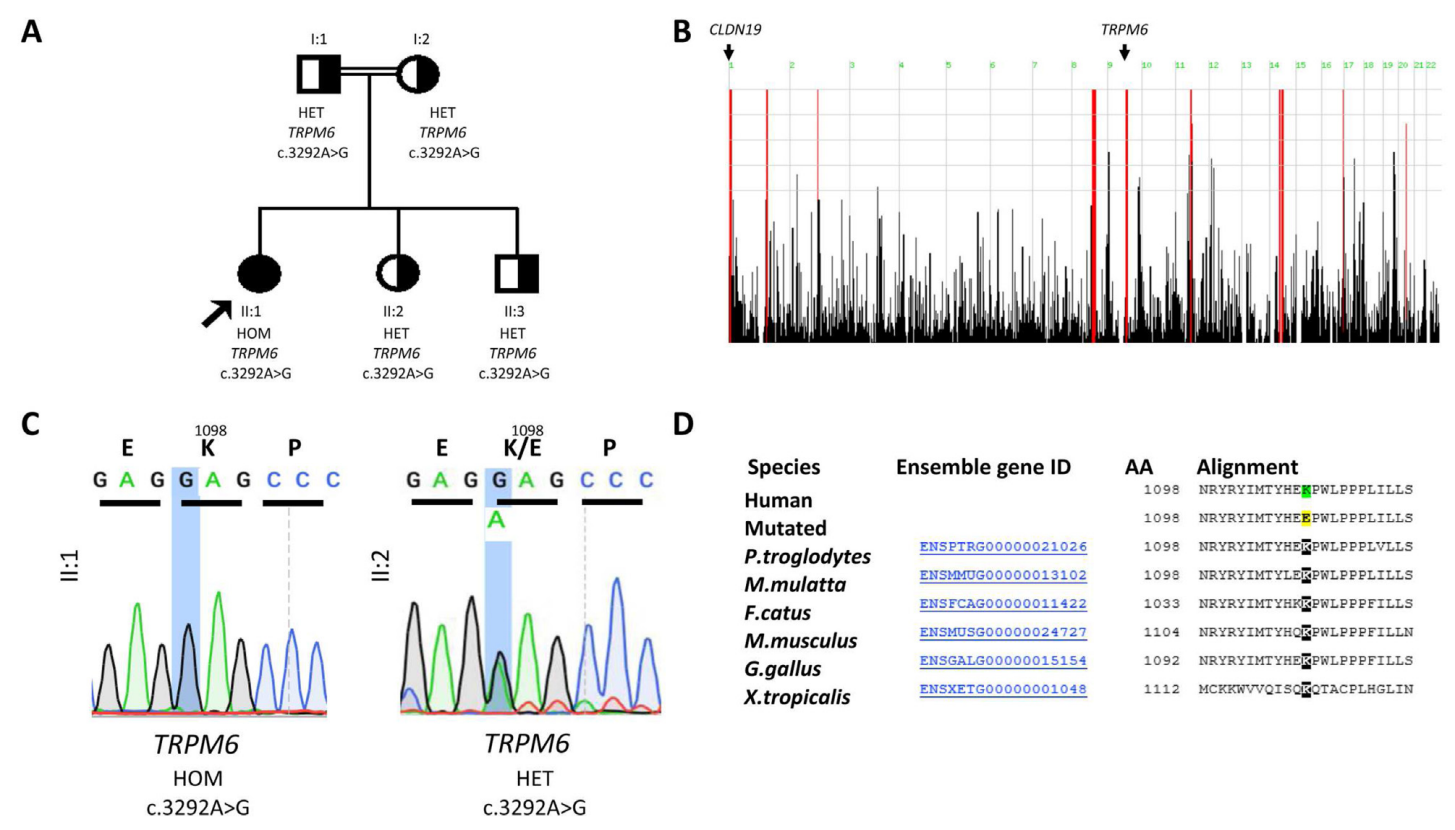
Identification of *TRPM6* missense mutation as the cause of familial hypomagnesaemia. ( **A**) Pedigree diagram showing proband (arrowed) who is homozygous for c.3292A>G p.(K1098E) missense mutation in TRPM6 and segregation of alleles from each parent. Parents were consanguineous (second cousins). ( **B**) Homozygosity mapping across chromosomes 1-22. Red bars indicate regions of homozygosity. Candidate genes *CLDN19* and *TRPM6* (arrowed) are located within regions of homozygosity. ( **C**) Chromatograms from proband (II:1) and sibling (II:2) showing *TRMP6* variant in homozygous and heterozygous state, respectively. ( **D**) Amino acid (AA) alignment showing conservation of the K1098 residue of *TRPM6*.

**Figure 2.**
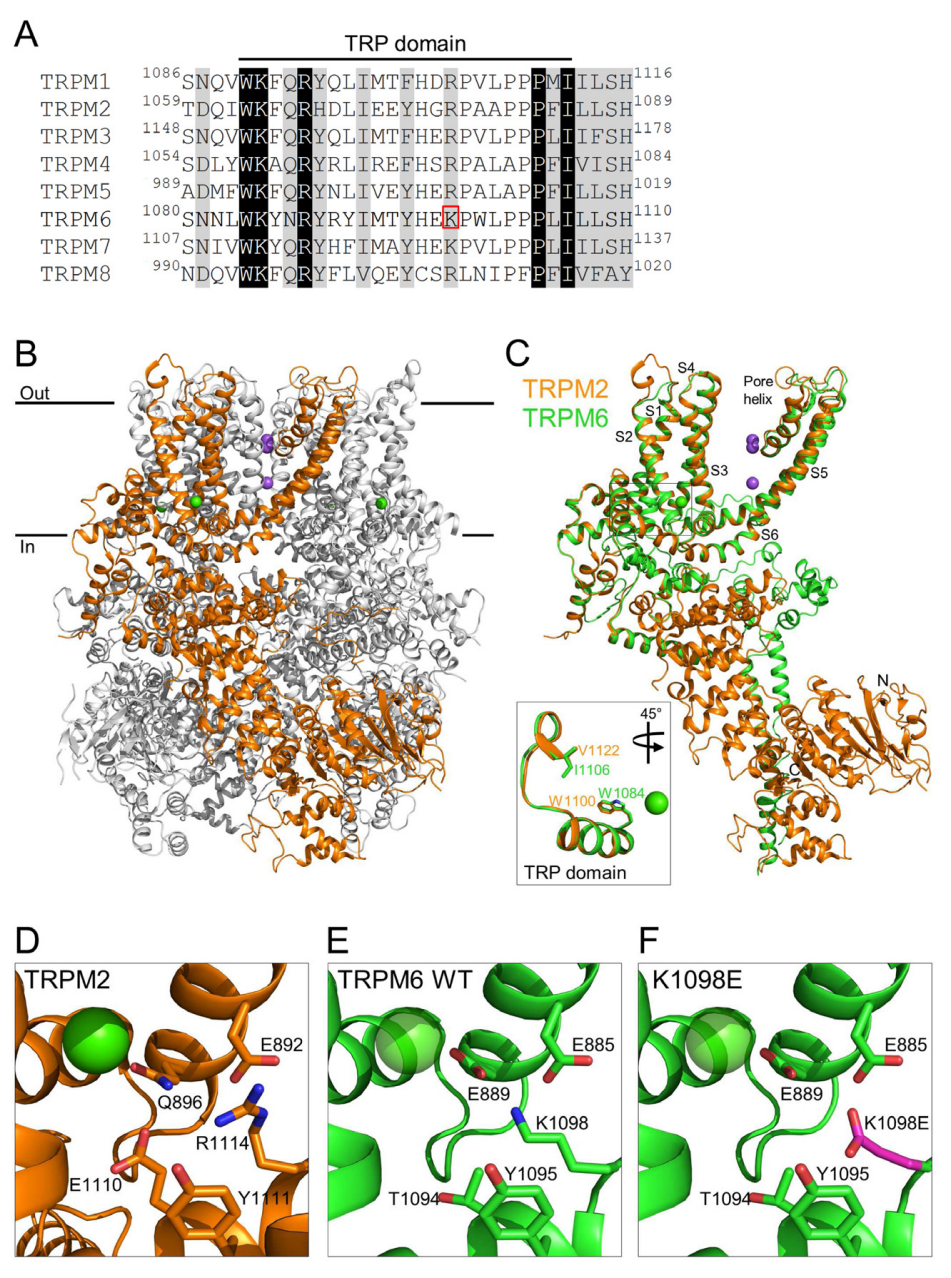
The TRPM6 K1098E variant affects a conserved basic residue in the TRP domain. ( **A**) Sequence alignments of TRP domain residues from human TRPM1-TRPM8. Fully-conserved residues are highlighted in black and semi-conserved residues in grey. TRPM6 K1098 is outlined in red. ( **B**) Cryo-EM structure of Ca ^2+^- and Na ^+^-bound TRPM2 (PDB 6CO7). One monomer of the homotetrameric channel is highlighted in orange and the co-ordinated Ca ^2+^ and Na ^+^ ions are shown as green and purple spheres, respectively. ( **C**) Superposition of the TRPM2 monomer (orange) and TRPM6 homology model (green). The TRP domain region (box and inset), wherein the TRPM6 K1098E variant lies, is shown to highlight the predicted structural homology between TRPM6 and TRPM2. The Ca ^2+^ ion (green sphere) is shown for orientation. ( **D**) Close up view of TRPM2 Q896 and E1110 involved in co-ordination of the Ca ^2+^ ion (green sphere) and R1114 (homologous to TRPM6 K1098, shown in ( **E**)) predicted to form a stabilising interaction within the TRP domain. ( **F**) Mutation of TRPM6 K1098 to glutamate (magenta) is predicted to disrupt stabilising TRP domain interactions. The relative position of the Ca ^2+^ ion (green sphere) in TRPM2 is shown in ( **E**) and ( **F**) for orientation.

Detailed information on the techniques described below is given in the *Methods* section. Following informed consent, whole exome sequencing (WES) was performed in the eldest sibling, II:1 ( Figure 1). Analysis using a combination of homozygosity mapping and variant calling revealed a homozygous missense mutation c.3292A>G, p.(K1098E) in *TRPM6* within a large region of homozygosity by descent ( Figure 1). The missense variant was confirmed by Sanger sequencing, and cascade screening confirmed this variant was in its heterozygous state in both parents and both mildly affected siblings. *In silico* tools confirmed evolutionary conservation ( Figure 1) as well as the rarity and predicted pathogenicity of the variant ( Table 2). Using predictive modelling of the protein structure we were able to show that the lysine residue at position 1098 is predicted to form a stabilising interaction within the TRP domain, and that the missense mutation of TRPM6 K1098 to glutamate is predicted to disrupt this interaction ( Figure 2).

**Table 2.** TRPM6 variant and *in silico* analysis.

Gene	Nucleotide variant ^#^	Predicted amino acid change	ExAC frequency	gnomAD	MutationTaster	SIFT	POLYPHEN2
*TRPM6*	c.3292A>G	p.(K1098E)	Not detected	Not detected	Disease causing	Deleterious	Possibly damaging

^# ^Reference sequence NM_017662.5

## Discussion

As the second most abundant intracellular cation, magnesium is vital for normal cell function ^1^. The majority of ingested magnesium load is absorbed in the distal small bowel via paracellular mechanisms, and the remainder is absorbed in the colon by transient receptor potential melastatin type 6 (TRPM6) ion channels in gut epithelium ^1^. Serum magnesium levels make up a relatively tiny proportion of whole-body magnesium content, but need to be kept within a narrow range to maintain neuronal, skeletal muscle and cardiac muscle cell stability. Serum magnesium homeostasis is therefore tightly regulated by reabsorption in the kidney; the majority is reabsorbed in the thick ascending limb of the loop of Henle via a paracellular route, and the ‘fine-tuning’ is performed in the distal convoluted tubule (DCT) via apically located TRPM6 channels.

Hypomagnesaemia is a common electrolyte disturbance, with a prevalence of 20% in hospitalised patients ^6^. Causes in adults include inadequate intake, refeeding syndrome, renal losses, gastrointestinal losses in diarrhoea, gastrointestinal malabsorption, and medications such as proton pump inhibitors (PPIs) ^7^. Serum magnesium levels may be requested as part of an extended biochemical panel if there is clinical concern about these risk factors, if symptoms or cardiac arrhythmia are present, or if other disturbances such as hypokalaemia or hypocalcaemia prompt the consideration of magnesium depletion. Measurement of urinary magnesium may help distinguish between gastrointestinal and renal losses. Urinary magnesium levels will be low if hypomagnesaemia is secondary to gastrointestinal losses, as the kidneys appropriately work to maximally reabsorb filtered magnesium, but raised or inappropriately normal despite low serum magnesium levels in renal magnesium wasting conditions. The majority of renal causes of hypomagnesaemia are not genetic, such as renal losses induced by post-obstructive diuresis, the recovery phase of acute tubular necrosis, hypercalcaemia, or drugs such as loop and thiazide diuretics, cisplatin, tacrolimus and aminoglycosides. 

Magnesium wasting disorders found in families have been shown to be associated with over a dozen genes ^5^. Similar to other monogenic diseases causing renal tubule phenotypes, the study of these diseases has greatly contributed to our knowledge of the renal tubular transport proteins responsible for homeostatic and physiological functioning. Familial hypomagnesaemic renal disorders may be inherited in both autosomal dominant and recessive patterns, and the underlying genes uncovered so far all encode proteins found in the thick ascending limb of the loop of Henle or DCT. Familial hypomagnesaemias may be categorised into four groups. These include hypercalciuric hypomagnesaemias (secondary to mutations in *CLCNKB* (Bartter syndrome type 3), *CLDN16*, *CLDN19*, *CASR*); Gitelman-like hypomagnesaemias (secondary to mutations in *SLC12A3* (Gitelman syndrome), *BSND* (Bartter syndrome type 4), *KCNJ10*, *FXYD2*, *HNF1B*, *PCBD1*); mitochondrial hypomagnesaemias; (mutations in *SARS2*, *MT-TI* and Kearns–Sayre syndrome) and other hypomagnesaemias (secondary to mutations in *TRPM6*, *CNNM2*, *EGF*, *EGFR*, *KCNA1*, *FAM111A*, *ATP1A1*) ^5^. De novo mutational events, in addition to recessive and dominant mutations, are also a possibility, especially for CNNM2 and ATP1A1.

*TRPM6* is expressed in both the colon and the DCT of the kidney, and ** mutations here can cause the condition known as hypomagnesaemia with secondary hypocalcaemia (HSH, OMIM 602014). There have been dozens of distinct mutations in *TRPM6* associated with this condition, and different variants can cause different effects on the function of the TRPM6 transporter ^1^. In patients with *TRMP6* mutations magnesium absorption from the colon is decreased (primary intestinal hypomagnesaemia), and the DCT is unable to perform the ‘fine-tuning’ of magnesium reabsorption and inappropriately wastes magnesium via the urine. Due to this dual pathology, the condition can cause the most profound electrolyte wasting of the genetic hypomagnesaemias. It typically presents in the neonatal or the infancy period (ranging from a couple of days to 8 months, rarely later) with severe symptoms due to hypomagnesaemia and hypocalcaemia such as seizures, which are subsequently responsive to magnesium administration ^8^. The hypocalcaemia is thought to be secondary to hypoparathyroidism, which is induced by hypomagnesaemia ^9^. Interestingly, the observation that treatment with PPIs is associated with hypomagnesaemia has led to a proposed mechanism of PPI-induced inhibition of TRPM6 and TRPM7 channels in the gastrointestinal tract ^10^. TRPM6 may also be downregulated in the DCT in response to cyclosporine, resulting in renal magnesium wasting ^11^.

Treatment of all the genetic hypomagnesaemia disorders, including those caused by *TRPM6* mutations, is with magnesium replacement therapy, either oral or intravenous depending on urgency and the tolerability of oral products. The major side-effect of oral magnesium replacement is diarrhoea, which can limit treatment compliance and paradoxically cause worsening of hypomagnesaemia due to increased gastrointestinal losses. Overall the prognosis of hypomagnesaemia with secondary hypocalcaemia is excellent, and serum calcium levels normalise as serum magnesium levels improve.

Given what is known about HSH, our first patient presented typically, with severe symptoms and the expected biochemical profile, including low PTH. WES confirmed a homozygous missense mutation in *TRPM6*, and clearly the family history of consanguinity was consistent with the diagnosis of an autosomal recessive disorder. In keeping with previous case reports she did not maintain magnesium concentration in the normal range, despite high dose oral replacement.

HSH is thought to represent a classic autosomal-recessive disease, with unaffected heterozygous parents and siblings. Interestingly in this case, the second child also had severe symptoms at presentation despite ultimately proving to be heterozygous for the *TRPM6* mutation. However, it can be seen that her presentation was less fulminant, without the development of tonic-clonic seizures and with milder derangement of biochemical parameters. In keeping with this less severe phenotype, she now maintains normal serum magnesium levels without supplementation. Finally, the third sibling had documented transient and asymptomatic hypomagnesaemia, which corrected by 1 year of age. These two siblings provide some evidence that a heterozygous allele in infants may lead to a transient biochemical phenotype, presumably related to the immaturity of the DCT to regulate magnesium. Such a finding has not been previously reported. It is possible this represents a mutation specific phenotype, rather than a general finding in patients with heterozygous TRPM6 mutations. Adults heterozygous for *TRPM6* pathogenic variants have never been reported to have abnormal serum magnesium levels ^12^. Heterozygous *Trpm6* knockout mice exhibit mild hypomagnesaemia under a normal diet, suggesting that a milder phenotype may be associated with the loss of one *TRPM6* allele ^13^. Newborns usually start with their mothers serum magnesium at birth, and if defective TRPM6 present then show a continuous decline over subsequent weeks. Some advocate that measurement of serum magnesium can exclude a disease state in siblings, but in this case it is possible that this would have suggested a diagnosis of HSH in the heterozygous siblings given their transient phenotype. It our knowledge the discovered p.K1098E (Lys1098Glu) variant found in this family is the first missense mutation described directly affecting the TRP domain of the TRPM6 ion channel subunit. The vast majority of previously reported TRPM6 mutations are nonsense, including stop mutation or small deletions or insertions ^12^, and only a small number of missense mutations have been reported and even fewer had a functional assessment.

**Figure 3.**
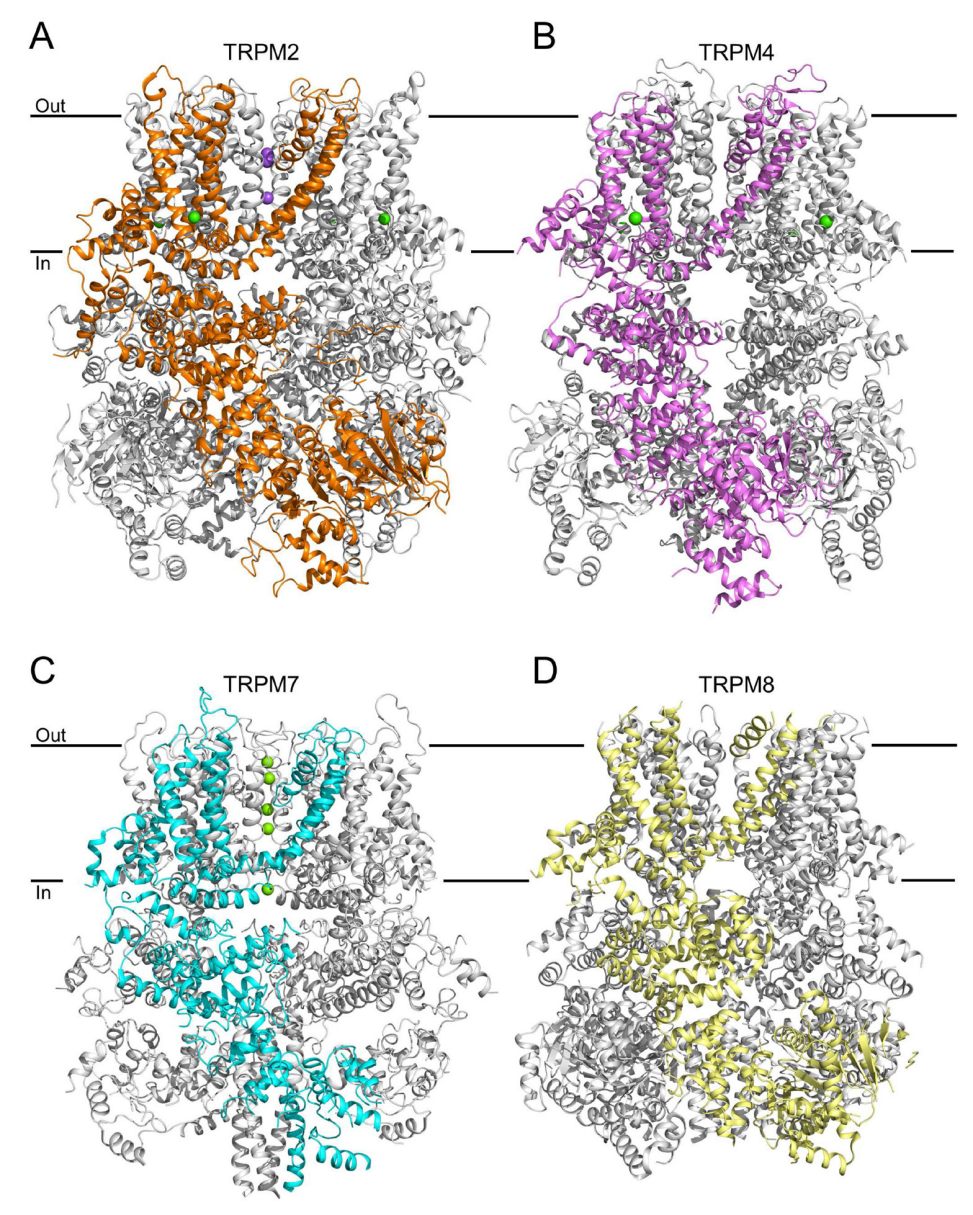
TRPM family members exhibit a conserved global structural architecture. Cryo-EM structures of ( **A**) TRPM2 (PBD 6CO7), ( **B**) TRPM4 (PDB 6BQV), ( **C**) TRPM7 (PDB 6BWD) and ( **D**) TRPM8 (PDB 6BPQ). One monomer of the respective tetrameric channel structure is highlighted. Spheres denote: Ca ^2+^ (green) and Na ^+^ (purple) ions in TRPM2; Ca ^2+^ ions (green) in TRPM4; and Mg ^2+^ ions (green) in TRPM7.

The location and predicted pathological effect of the missense mutation warrants further discussion. Previously described missense mutations in *TRPM6* include p.(S141L) and p.(P1017R), which lead to either trafficking or gating impairment of the TRMP6 channel ^14, 15^. Additional missense mutations p.(I174R), p.(T354P) and p.(C707T) have also been reported ^16^. Here we have taken advantage of recently published cryo-EM structures of TRPM2 ^17– 19^, TRPM4 ^20– 23^, TRPM7 ^24^ and TRPM8 ^25^, which suggest a conserved global architecture for TRPM family members, consistent with sequence conservation (see *Extended data*, Supplementary Figure 1) ^26^. We therefore utilised all structures to interpret the likely pathogenic effect of the TRPM6 K1098E variant (with the exception of TRPM8, due to low resolution in the homologous region ^25^). TRP domain sequence analysis ( Figure 2A) revealed conservation of a basic residue at the homologous position to K1098 in TRPM6, indicating an important functional role for the positively charged side-chain. Studies of TRPM6-TRPM8 suggest that positively charged residues in the TRP domain may interact with the negatively charged phosphate groups in phosphatidylinositol-4,5-bisphophate (PIP _2_) to mediate channel activation ^27, 28^. Neutralisation of the positive charge by substitution with glutamine was shown to abolish channel activity in TRPM6 K1098Q ^27^ and TRPM8 R1008Q ^28^, although surprisingly no significant effect on channel activity was seen in the homologous TRPM7 K1125Q variant ^27^. Moreover, the homologous TRPM4 variant (R1072Q) exhibited normal sensitivity to PIP _2_, arguing against this residue being involved directly in PIP _2_ binding ^29^. Based on the available TRPM structures, we predict K1098 may mediate one of several stabilising interactions in TRPM6. In the cryo-EM structure of TRPM2 ( Figure 2B), and TRPM6 homology model ( Figure 2C), the TRP domain lies in close proximity to the ion conduction pathway, with mutations in this domain likely to affect channel gating. Indeed, mutation of TRP domain residues in TRPM2 (E1110) and TRPM4 (E1068) were shown to impair the binding of Ca ^2+^ necessary for priming the channel for voltage-dependent opening ^19, 20, 30^. In TRPM2, E1110 in the TRP domain stabilises Q896 in the S2 helix ( Figure 2D), correctly orienting Q896 for Ca ^2+^-binding ^19^. Interestingly, the glutamine residues involved in co-ordination of the Ca ^2+^ ion in TRPM2 (Q896) and TRPM4 (Q831 ^20^) are conserved in all Ca ^2+^-dependent TRPM channels ( Figure 3), but are replaced by glutamate (E889) in the Ca ^2+^-independent TRPM6 ( Figure 2E). TRPM6 modelling suggests that K1098 in the TRP domain could form stabilising interactions with E889 and E885 (equivalent to R1114 and E892 in TRPM2 ^19^; Figure 2D) in the S2 helix ( Figure 2E), thereby priming the channel for activation in a Ca ^2+^-independent manner. Alternatively, TRPM6 K1098 may potentially serve to stabilise the TRP domain helix itself, either through a cation-π interaction with Y1095 ( Figure 2E), equivalent to that identified in the cryo-EM structure of TRPM4 between R1072 and F1069 ^22^, or via interaction with the hydroxyl side-chain of T1094 (equivalent to TRPM2 E1110; Figure 2E). This latter potential interaction is analogous to that modelled between TRPM8 R1008 and E1004, whereby agonist/antagonist binding was predicted to modulate the position and intra-protein contacts of R1008 (equivalent to TRPM6 K1098), with resultant changes in the TRP domain helix effecting channel opening/closing, respectively ^31^. Substitution of TRPM6 K1098 with the negatively charged glutamate (K1098E; Figure 2F) is predicted to be pathogenic since this change would destabilise any of the potential interactions discussed.

## Conclusion

Here we provide evidence for a novel pathogenic missense mutation p.(K1098E) in *TRPM6* which leads to a severe hypomagnesaemia with secondary hypocalcaemia phenotype in an affected child. *In silico* modelling of homologs of the TRPM channels supports an important stabilising role for this residue.

## Methods

Clinical summaries were prepared and DNA samples taken from whole blood following informed and written consent. Ethical approval for this study was obtained from the National Research Ethics Service (09/H0903/36).

A DNA sample from the affected proband underwent WES, performed via GATC Biotech. A DNA library was prepared using enrichment with SureSelectXT and a human All Exon Kit. Sequencing was performed using Illumina with paired end reads of 2 ×150 bp with a >30X average on target coverage. Raw data was analysed via a commercial bioinformatics pipeline (GATC Eurofins), which included mapping against genomic reference sequence and detection of SNPs and InDels using GATK’s Haplotype caller ^32^. Resulting vcf files were analysed using Qiagen Ingenuity Variant analysis software (Build 5.5.20190412) (or open access equivalent VCF-Explorer 1.0) and HomozygosityMapper.

Variants in genes and segregation in other family members were confirmed using exon PCR followed by Sanger sequencing.

Human TRPM6 (UniProt accession Q9BX84) was modelled against the cryo-EM structures of TRPM2 (PDB accession 6CO7; ^19^), TRPM4 (PDB 6BQV; ^20^ and TRPM7 (PDB 6BWD; ^24^ using HHPred ^33^, Modeller (version 9.21) ^34^ and I-TASSER ^35^ software. TRPM structure figures were prepared using PyMOL 2.3.

## Data availability

### Underlying data

Whole exome sequencing data available from BioProject, accession number PRJNA541906: https://identifiers.org/ncbi/bioproject:PRJNA541906.

### Extended data

Figshare: Alignment of human TRPM channels 1-8 amino acid sequences. https://doi.org/10.6084/m9.figshare.8063537 ^26^.

This project contains the following extended data:

- **Supplementary Figure 1. Alignment of human TRPM channels 1-8 amino acid sequences (UniProt accession codes Q7Z4N2, O94759, Q9HCF6, Q8TD43, Q9NZQ8, Q9BX84, Q96QT4 and Q7Z2W7, respectively), with sequences for the cryo-EM structures of *N. vectensis* (Nv) TRPM2 (UniProt and mouse (Ms) TRPM7 (UniProt Q923J1).** The position of the TRP domain, TRPM6 K1098 and homologous residues are highlighted.

Extended data are available under the terms of the Creative Commons Attribution 4.0 International license (CC-BY 4.0).

## Consent

Written informed consent was obtained from the patients’ family for publication of this case report and accompanying images.
