# Supplementary material for: Case Report: Investigation and molecular genetic diagnosis of familial hypomagnesaemia
Source: F1000Res. 2019 Dec 5;8:666. Originally published 2019 May 15. [Version 2] doi: 10.12688/f1000research.19006.2 (PMC6694456; doi:10.12688/f1000research.19006.2)
Supplement: Peer review history

# Peer review: Reviewer response for version 2

**DOI:** 10.5256/f1000research.23651.r57481

**Published:** 2020-02-14

**Authors:** Karl Peter Schlingmann

**Affiliations:** 1 Department of General Pediatrics, University Children’s Hospital, Münster, Germany

The critizisms raised during the first Review process have been carefully addressed by the authors. Especially, the information on the maternal serum magnesium level has been added, and the milder hypomagnesemia in the two heterozygous siblings is discussed in more detail now. Also, the possibility of both, a transient phenomenon as well as a mutation-specific effect is discussed.

 Finally, the minor comments and suggestions have been implemented.

 From my point of view, the manuscript in its present form is suitable for publication!

I confirm that I have read this submission and believe that I have an appropriate level of expertise to confirm that it is of an acceptable scientific standard.

# Peer review: Reviewer response for version 2

**DOI:** 10.5256/f1000research.23651.r57483

**Published:** 2020-02-11

**Authors:** Abhijit Dixit

**Affiliations:** 1 Department of Clinical Genetics, Nottingham University Hospitals NHS Trust, Nottingham, UK

The authors have addressed most of the questions raised in the review in their revised version.

I confirm that I have read this submission and believe that I have an appropriate level of expertise to confirm that it is of an acceptable scientific standard.

# Peer review: Reviewer response for version 2

**DOI:** 10.5256/f1000research.23651.r57484

**Published:** 2020-01-21

**Authors:** Lilia Romdhane

**Affiliations:** 1 Laboratory of Biomedical Genomics and Oncogenetics, Pasteur Institute of Tunis, University of Tunis El Manar, Tunis, Tunisia; 2 Faculty of Sciences, Bizerte, University of Carthage, Zarzouna, Tunisia

Please take into consideration my previous comments. In addition, is incomplete penetrance of variants in genes involved in hypomagnesaemia has been reported?

I confirm that I have read this submission and believe that I have an appropriate level of expertise to confirm that it is of an acceptable scientific standard, however I have significant reservations, as outlined above.

# Peer review: Reviewer response for version 1

**DOI:** 10.5256/f1000research.20831.r48695

**Published:** 2019-08-09

**Authors:** Na Zhu

**Affiliations:** 1 Department of Systems Biology, Columbia University Medical Center, Columbia, USA

The authors identified that a novel deleterious missense variant is causal to hypomagnesemia. They found this variant from a pediatric patient in consanguineous family. Both parents are heterozyous carriers while the patient is homozygoty carrier. They inferred the pathogenicity of this variant using *in silico* method.

 This variant is never seen in any large population datasets, it is predicted to be deleterious using various prediction tools. This variant is also indicated disrupting stabilising TRP domain from the protein structure analysis tool. The call is also verified to be true using chromatogram. Most importantly, TRPM6 has been reported to be associated with hypomagnesemia. Therefore, I agree with the authors conclusion, this variant is a causal variant to this patient.

I confirm that I have read this submission and believe that I have an appropriate level of expertise to confirm that it is of an acceptable scientific standard.

# Peer review: Reviewer response for version 1

**DOI:** 10.5256/f1000research.20831.r50544

**Published:** 2019-07-15

**Authors:** Abhijit Dixit

**Affiliations:** 1 Department of Clinical Genetics, Nottingham University Hospitals NHS Trust, Nottingham, UK

In this manuscript, Jamie Willows and colleagues describe the genetic diagnosis of hypomagnesemia with secondary hypocalcemia (HSH) in a consanguineous family, with identification of a novel homozygous TRPM6 missense variant in the proband. The authors have done a good job of placing this rare condition in the context of wider genetic and acquired causes of hypomagnesemia. There is a fair degree of detail in terms of the clinical course and investigations - see comments below for suggested changes.

 The identification of a *missense* variant is noteworthy. The authors do not highlight the fact that the vast majority of previously reported TRPM6 mutations result in loss-of-function of the protein. Only a small number of missense mutations have been reported and still fewer have had functional assessment - still showing loss of TRPM6 channel function (Lainez *et al.*, 2014 ^1^). Functional studies are, however, not easy to undertake and the authors have provided evidence supporting pathogenicity of the novel variant in their family using protein modelling.

 The most interesting and possibly contentious issue is the identification of neonatal hypomagnesemia in the siblings with heterozygous p.Lys1098Glu variant. My understanding from the literature is that even asymptomatic hypomagnesemia has not been documented in adult carriers of TRPM6 mutations, and therefore symptomatic presentation would be very unusual indeed. The authors attribute the hypomagnesemia in the heterozygous siblings to immaturity of the DCT. The following points, in my view, merit some extra thought:

1. The only serum magnesium value provided for the sibling II:2 is 0.53mmol/L in the context of ‘abnormal eye movements.’ The complex partial seizure is not described and no further definite seizure episode is documented. Is it possible to be certain that this episode represented a symptomatic seizure and suggest a causal relationship with modest hypomagnesemia? Were other investigations (e.g. MRI brain, blood sugar etc.) performed to identify an alternative explanation for the child's paroxysmal episode?
2. Information on magnesium levels in the parents would be very important and, if possible, maternal magnesium levels around the time of delivery.
3. It is not specified for how long the magnesium supplementation was prescribed in the two children.
4. It would be helpful to clarify the timeline of events further. Before exome sequencing revealed the diagnosis, what were the possible diagnostic considerations in the two older siblings?

Lastly, some minor changes would further improve the manuscript:

  

1. Three letter amino acid nomenclature is preferable to the single letter codes now, i.e. Lys1098Glu instead of K1098E.
2. In Table 1, the current magnesium levels of patient II:1 has a decimal point missing. ‘0.4-0.6mmol/L’ is presumably what was intended.
3. It is preferable to say ‘second cousins’ rather than ‘second-degree cousins’, as family relationships in degree terms implies parents/siblings and children as first degree relatives and uncles/grandparents as second degree relatives. First cousins would be third degree relatives. Ideally, a proper pedigree should be drawn so that the familial relationship is clear.

I confirm that I have read this submission and believe that I have an appropriate level of expertise to confirm that it is of an acceptable scientific standard, however I have significant reservations, as outlined above.

## References

[1] : New TRPM6 missense mutations linked to hypomagnesemia with secondary hypocalcemia. *Eur J Hum Genet*.2014;22(4) : 10.1038/ejhg.2013.178 497-504 10.1038/ejhg.2013.178 23942199PMC3953905

# Peer review: Reviewer response for version 1

**DOI:** 10.5256/f1000research.20831.r50062

**Published:** 2019-07-03

**Authors:** Lilia Romdhane

**Affiliations:** 1 Laboratory of Biomedical Genomics and Oncogenetics, Pasteur Institute of Tunis, University of Tunis El Manar, Tunis, Tunisia; 2 Faculty of Sciences, Bizerte, University of Carthage, Zarzouna, Tunisia

The authors provide a molecular investigation of a familial hypomagnesaemia using whole exome sequencing in a consanguineous family from Oman. A novel missense mutation in the *TRPM6* gene at homozygous state has been identified in the index patient. A bioinformatic analysis using a modelling approach was performed to explain the pathogenic effect of the novel mutation.

**General comments:**

 The authors have to provide the OMIM numbers of the diseases reported in the manuscript. The version of the human genome used for read alignment and annotation also has to be mentioned. The status of the identified mutation “novel or new” is only provided in the abstract and the conclusion. Is this family the first one reported with the disease from Oman? The result section drastically lacks details. In addition, details on the methods and tools used during this study have to be provided. Please divide the “Methods” section into sub-sections: Example: Whole Exome Sequencing, WES data analysis, Homozygosity mapping, TRMP6 ortholog alignment, Structural modelling …

**Specific comments:**

**Question:** Are enough details provided of any physical examination and diagnostic tests, treatment given and outcomes?

**Reviwer’s comment:** Clinical description of the parents are lacking as it is mandatory to explain why heterozygous siblings show a moderate phenotype and therefore, helps the interpretation of the genetic results. Are the parents also showing abnormal level of magnesium ?

**Question:** Is sufficient discussion included of the importance of the findings and their relevance to future understanding of disease processes, diagnosis or treatment?

**Reviwer’s comment:** Familial hypomagnesaemia (OMIM#602014) is a genetic autosomal recessive disorder caused by mutations in the *TRPM6* gene in homozygous or compound heterozygous state. The authors identified a novel missense mutation at homozygous state in the index patient. The parents and the sister and brother were heterozygous. In the absence of the precise clinical description of the parents, the finding of the expression of some clinical symptoms in the brother and sister harboring the mutation at the heterozygous state is not consistent with the autosomal recessive transmission mode of the disease. Therefore, are the parents completely healthy or are they showing some hypomagnesaemia? The authors state that no adults heterozygous for *TRPM6* mutations have been reported showing abnormal serum magnesium levels. Is this statement supported by any biochemical dosages in the parents of the studied family? As heteroygous carriers with moderate expression of an autosomal recessive disease have been reported in a consanguineous population (Mokni et al ^1^) the authors have to provide further clinical details of the parents and reformulate their hypothesis.

 Moreover, as the authors performed a whole exome sequencing, they have access to the completed catalogue of coding variants of the patient. Is the patient harboring other variants of functional effects in the *TRPM6* gene and/other candidate genes? in modifier genes? Are these variants present in the brother and sister that help explaining their phenotypes?  Lainez et al ^2^, reported a patient with a *TRPM6* heterozygous mutation with familial hypomagnesaemia. They explained the phenotype by the presence of a variant, that when combined with the pathogenic mutation, drastically decreases the magnesium level. They supported they statement with functional assay. Therefore, the authors are invited to perform again the bioinformatic analysis of variant annotation and filtering. Consequently, both the result and discussion sections have to be revised.

I confirm that I have read this submission and believe that I have an appropriate level of expertise to confirm that it is of an acceptable scientific standard, however I have significant reservations, as outlined above.

## References

[1] : Heterozygous manifestations in female carriers of Mal de Meleda. *Clin Genet*.2004;65(3) :244-6 1475667610.1111/j.0009-9163.2004.00224.x

[2] : New TRPM6 missense mutations linked to hypomagnesemia with secondary hypocalcemia. *Eur J Hum Genet*.2014;22(4) : 10.1038/ejhg.2013.178 497-504 10.1038/ejhg.2013.178 23942199PMC3953905

# Peer review: Reviewer response for version 1

**DOI:** 10.5256/f1000research.20831.r48551

**Published:** 2019-06-24

**Authors:** Karl Peter Schlingmann

**Affiliations:** 1 Department of General Pediatrics, University Children’s Hospital, Münster, Germany

Dear editors and authors,

 In their manuscript entitled "Investigation and molecular genetic diagnosis of familial hypomagnesemia: a case report” Jamie Willows and colleagues describe a patient with the typical clinical presentation of familial hypomagnesemia with secondary hypocalcemia (HSH). Molecular genetic studies identified a homozygous mutation in the *TRPM6* gene as the underlying pathology. The TRPM6 gene encodes a member of the transient receptor potential (TRP) family of ion channels that is involved in the formation of epithelial magnesium permeable ion channels in intestine and kidney. If it does so alone or in cooperation with TRPM7 has been a matter of debate.   

 In addition to the initial studies and two larger follow-up reports, ^1^^, ^^2^^,^^3^^, ^^4^ mostly small case series or case reports have been published of patients with HSH and mutations in *TRPM6*. HSH is thought to represent a classic autosomal-recessive disease with unaffected heterozygous parents and siblings. Most patients were found to carry non-sense mutations in TRPM6 including stop mutations, small deletions/insertions, leading to a shift in the reading frame and premature stops of translations, splice site mutations, and also deletions of larger parts of the gene. Only a small number of missense mutations have been reported of which a subset has been analyzed functionally. ^4^^, ^^5^^, ^^6^ These almost uniformly lead to a complete loss-of-function.           

 Study design and results of this report are presented accurately and the appropriate literature is cited correctly. The methods used for molecular diagnosis and in-silico modeling of the identified mutant are provided. The initial presentations as well as diagnostic tests performed, treatments given and clinical outcomes of the index patient and siblings are described in sufficient detail. In the context of the published literature, this case report is unique for two reasons: to my knowledge, the discovered p.K1098E variant is the first missense mutation directly affecting the TRP domain of the TRPM6 ion channel subunit. The TRP domain is thought to play a crucial role in ion channel multimerization as well as in channel activation by PIP2 (phosphatidylinositol-4,5-bis-phosphate). Accordingly, the authors present comprehensive data analyzing the putative effects of the discovered mutant by comparing this naturally occurring mutant to engineered mutants at the identical position in TRPM6 and related TRPM channel subunits. Though the effects of engineered mutants on channel activity as well as PIP2 mediated channel activation are not consistent, the genetic data presented here together with the disease phenotype clearly argue for the pathogenetic role of the discovered mutant. It will be interesting to study this mutant functionally in an overexpression system (especially in combination with wildtype TRPM6 subunits and TRPM7, see below). The second intriguing finding presented here is that, in addition to the index patient, two siblings carrying the p.K1098E variant in heterozygous state presented in infancy with hypomagnesemia and a cerebral seizure in case of one sibling. Such a finding in heterozygous mutation carriers has not been reported before. The authors consider this finding a general feature of the disease and attribute it to a possible immaturity of the renal tubule in early life. Serum magnesium levels have not been systematically evaluated in clinically unaffected siblings so far. However, measurements of serum magnesium levels in the first weeks of life have been advocated in siblings to exclude disease before clinical presentation with cerebral seizures and before quick genetic testing became feasible. Newborns usually start with their mothers serum magnesium at birth and in case of defective TRPM6 show a continuous decline of serum levels over the following weeks. At least in single families, measurement of serum magnesium yielded normal levels and was able to exclude disease in younger siblings (unpublished data, personal observation). Therefore, the information on maternal serum magnesium is critical in this family to better classify the observed changes in the heterozygous siblings as following the mentioned approach, the measurement of serum magnesium levels would have suggested the diagnosis of HSH also in the heterozygous siblings of this family rather than excluding classic disease. Finally, it is also conceivable that the hypomagnesemia observed in the siblings of the family presented here represents a mutation specific phenomenon rather than a general finding in patients with heterozygous TRPM6 mutations which could also be discussed in the report. Are there indications of a functional effect of the p.K1098E mutant on heteromultimerization with wildtype TRPM6 or TRPM7 via TRP domain interactions? Were there additional variants in the TRPM6 gene identified by WES (especially on the unaffected alleles of siblings)? Do the two siblings share the identical unaffected allele of mother or father? Could an additional variant potentially explain the transient phenotype?

 Minor comments and suggestions:

- the dose of daily oral magnesium should please be provided in mmol/kg/day as it allows easier comparison with published doses (500mg Mg-sulphate should be 4.06mmoles)
- discussion line 7: “need” instead of “needs” (serum levels)
- could you please add a citation for the  physiological summary in the first discussion section (maybe Dai & Quamme, Phys Rev 2001? ^7^)
- page 6, second paragraph: FXYD2 instead of FYXD2, CNNM2 instead of CNMM2.
- page 6, second paragraph: I am not aware of inherited EGFR mutations?!
- page 6, second paragraph: ATP1A1 could be added.
- page 6, second paragraph: the possibility of de-novo mutational events (in addition to recessive and dominant) should be mentioned (especially for ATP1A1 and CNNM2).
- please provide OMIM number and nomenclature for HSH.
- page 6, third paragraph: patients typically present in the neonatal period and in infancy (ranging from a couple of days to ~8 months, rarely later)

I confirm that I have read this submission and believe that I have an appropriate level of expertise to confirm that it is of an acceptable scientific standard, however I have significant reservations, as outlined above.

## References

[1] : Hypomagnesemia with secondary hypocalcemia is caused by mutations in TRPM6, a new member of the TRPM gene family. *Nat Genet*.2002;31(2) : 10.1038/ng889 166-70 10.1038/ng889 12032568

[2] : Mutation of TRPM6 causes familial hypomagnesemia with secondary hypocalcemia. *Nat Genet*.2002;31(2) : 10.1038/ng901 171-4 10.1038/ng901 12032570

[3] : Novel TRPM6 mutations in 21 families with primary hypomagnesemia and secondary hypocalcemia. *J Am Soc Nephrol*.2005;16(10) : 10.1681/ASN.2004110989 3061-9 10.1681/ASN.2004110989 16107578

[4] : New TRPM6 missense mutations linked to hypomagnesemia with secondary hypocalcemia. *Eur J Hum Genet*.2014;22(4) : 10.1038/ejhg.2013.178 497-504 10.1038/ejhg.2013.178 23942199PMC3953905

[5] : Disruption of TRPM6/TRPM7 complex formation by a mutation in the TRPM6 gene causes hypomagnesemia with secondary hypocalcemia. *Proc Natl Acad Sci U S A*.2004;101(9) : 10.1073/pnas.0305252101 2894-9 10.1073/pnas.0305252101 14976260PMC365716

[6] : Hypomagnesemia with secondary hypocalcemia due to a missense mutation in the putative pore-forming region of TRPM6. *J Biol Chem*.2007;282(10) : 10.1074/jbc.M611117200 7656-67 10.1074/jbc.M611117200 17197439

[7] : Magnesium transport in the renal distal convoluted tubule. *Physiol Rev*.2001;81(1) : 10.1152/physrev.2001.81.1.51 51-84 10.1152/physrev.2001.81.1.51 11152754
